# King's College Hospital

**Published:** 1893-11-11

**Authors:** 


### The Institutional Workshop.

### WITHIN THE HOSPITALS.

### KING'S COLLEGE HOSPITAL.

(By Our Special Commissioner.)

King's College Hospital suffers from a great disadvantage, which the authorities are powerless to alter. Structurally it is behind the times. The wards for the most part are what is known as "back to back" wards, which prevents through ventilation, and although the superficial space per bed is unusually large, that in itself is not altogether an advantage. Indeed, we are of opinion that too much superficial space may be as great a fault in its way as too little. The ward kitchens and sculleries are inconveniently placed, and contain much more room than is either necessary or desirable. The lavatory and bath space is very limited, and altogether we believe this hospital may be regarded as the most difficult to administer, from a nursing point of view, of any institution in London. In such circumstances it is not easy to make the wards attractive, and a paucity of funds has no doubt prevented the supply of certain modern articles of furniture and fittings, which would tend to improve the appearance and lighten the work of the medical staff and nurses. All this, it must be understood, makes to the credit of the management, and the result of treatment in King's College Hospital at the present time, especially in view of the structural conditions, places Listerism upon the highest pinnacle of modern science. We cannot understand how it is that King's, which is or ought to be the hospital of the legal profession, should lack so many little embellishments which we believe the great body of the legal profession would consider it a privilege to supply, were the circumstances brought directly under their personal notice. A striking proof of the energy and spirit with which the present administration faces the difficulties with which it has to contend, is shown by the present condition of the nurses' quarters, on the top story of the hospital. Here will be found comforts equal to those of any other hospital, and an arrangement of cubicles for the sleeping accommodation of the nurses, which are distinctly the best ever planned for this purpose. The most inexperienced person who will take the trouble to visit King's College Hospital and inspect the wards and nurses' quarters, will see, by contrast, what might be done to improve the former, if only the money were forthcoming.

An Opening for Critical Givers.

Our experience teaches us that amongst those who contribute to hospitals, there exists a distinct class who object to invested funds and reliable income. They maintain that each generation should provide for its own sick, and that they therefore prefer to give their money to a hospital where they know the financial stress is so great as to ensure its expenditure upon the patients during the lifetime of the givers. They further hold the view that invested funds, by making the managers relatively independent of outside criticism, tend to lessen efficiency and promote abuses in other directions. We commend King's College Hospital to all who hold these views, because here they will find an institution which has no invested property, which depends absolutely upon voluntary contributions for its maintenance, and which therefore offers the maximum guarantee that every penny handed in to the hospital exchequer will be promptly expended in the relief of sickness and disease. We may further add that unless an adequate fund be forthwith raised for King's College Hospital (the committee appeal for £50,000 to constitute a five years' maintenance fund), great numbers of sick and suffering people must have their burden of misery materially increased. King's College requires an income of £16,500 per annum, and its annual subscription list amounts but to £2,000. It is therefore absolutely essential, if this hospital is to be continued, that the maintenance fund of £50,000 shall be forthcoming within the next few months. Having regard to the large number of poor persons who reside in the immediate neighbourhood of the hospital, to the splendid work which King's has done for science through its medical staff and school, and to the no less splendid work which it has done for nursing, formerly through St. John's House, and during the last ten years by its own direct agency under the able management of Miss Monk, this hospital is entitled to receive that full measure of public support which has been sadly wanting in the past. Its present financial position is a grievous reflection upon the legal profession, and we cannot but feel that if the facts are made fully known, the finances of King s College Hospital will speedily assume a condition adequate to its claims and circumstances.

Sir Joseph Lister's Hospital.

Apart from the claims which King's College Hospital has intrinsically upon the legal profession, and Londoners generally, its claims upon all who have benefited by surgical treatment during the last twenty years is even more paramount still. King's is Sir Joseph Lister's hospital, the scene of his triumphs and the field in which he has worked out successfully those grand principles of surgical practice which have made modern surgery the wonder of the world. Professors Simpson and Erichsen have shown, by statistics which have never been upset, that formerly the average mortality in town hospitals, after major surgical operations, ranged from 41·6 per cent. in 1868 to 37·8 per cent. in 1872 in the large metropolitan hospitals. Science never stands still; all true science is progressive. There is no finality of which we can be certain where science is concerned. Sir Joseph Lister's advent, and the introduction of the aseptic system has revolutionised the treatment of cases after surgical operations and of open wounds, both in hospital and private practice. Since the year 1878 Listerism has made rapid and convincing progress, and Dr. Schede has proved beyond dispute that Sir Joseph Lister, by his wonderful discovery, had enabled the surgeons who adopted it conscientiously, to reduce the mortality in such cases by the year 1880 to 4·36 per cent. To-day that mortality is considerably less, a fact which makes the financial condition of King's College Hospital at the present time a reflection upon the intelligence and common sense of the nation, We have shown that Sir

Joseph Lister's wonderful discovery has saved, on a low computation, 33 per cent. of that large class of patients who have had to undergo major surgical operations. Twenty years ago 33 persons out of every 100 who now recover after these operations were doomed to death Now, if we could collect the names of the thousands who still live and enjoy good health after operations, as the result of Sir Joseph Lister's work, we should find amongst them a large number of persons of both sexes who could give largely, and who ought to give freely, in support of King's College Hospital. Our words may, we hope, reach a large number of people who owe their lives, under Providence, to Sir Joseph Lister's teaching and work. We charge their consciences with the responsibility of longer refusing to look into the circumstances of King's College Hospital. If they accept the challenge, they must certainly enrol themselves as princely donors and supporters of an institution which has, in large measure, afforded the means which have enabled their benefactors to rescue them from the jaws of death and the bed of suffering. Such an appeal must prove irresistible to the most parsimonious of the race, though our experience teaches us that those who have suffered most have usually the most sympathy for the sufferings of their neighbours and friends. Let the authorities of King's College Hospital take up the suggestion here made, and follow it out with diligence until the £50,000 is forthcoming. If they pursue such a course, we make bold to say that the whole of the £50,000 should be safely lodged in the bank, to the credit of King's College Hospital, before the end of the present year.

### The Nursing and Convalescent Departments.

It was a fortunate day for King's College Hospital when Miss Monk was selected for the office of Sister-Matron. It is no reflection upon the many able ladies who preside over the nursing departments of our great hospitals to say that Miss Monk is one of the ablest, most zealous, and most capable matrons of the present day. Throughout her department there exists abundant evidence of the untiring energy, the trained intelligence, the technical knowledge, the kindly sympathy and the great organising powers which have combined to build up a character worthy of modern nursing, aye, and of England too. The difficulties which had to be faced ten years ago, owing to inadequate supply of linen, of accommodation, of staff and means, were so great as to require an exhibition of wisdom in the management which few possess. Ten years ago the nursing department of King's College Hospital left much to be desired. To-day its efficiency is widely recognised, and the system upon which it is managed exhibits points for imitation by most hospitals. Miss Monk has been ably seconded by her assistant, Sister Sibbald, whose powers as a teacher have enabled the probationers to acquire a sounder and fuller knowledge of nursing than falls to the lot of most nurses. The accommodation at King's for paying probationers is on the whole so good that 835 applications were received for admission to training during last year, although there were but thirty vacancies to fill. Not only are the nurses well trained, but they are trained upon a system which teaches them that it is a privilege for a nurse to undertake every duty required of her by the patient, without standing upon her dignity, or considering whether this or that is quite the class of work which a member of the nursing profession ought to undertake.

In addition to the nursing department, King's College is fortunate in the possession of a convalescent home at Hemel Hempstead, which admitted 463 patients during last year. This home possesses attractive architectural features, is situated in the centre of a most delightful country, and the patients have the advantage of a pony carriage, as well as the maximum opportunity for daily walks so as to enable them to enjoy the picturesque scenery. Miss Acheson, the Lady Superintendent, has given abundant proofs of the extent to which the comfort and happiness of the patients in a convalescent home depend upon the Lady Superintendent. Few convalescent homes are more popular than that at Hemel Hempstead, and few, if any, have done better work.

